# Gray Correlation Analysis and Prediction on Permanent Deformation of Subgrade Filled with Construction and Demolition Materials

**DOI:** 10.3390/ma12183035

**Published:** 2019-09-19

**Authors:** Junhui Zhang, Anshun Zhang, Jue Li, Feng Li, Junhui Peng

**Affiliations:** National Engineering Laboratory of Highway Maintenance Technology, Changsha University of Science & Technology, Changsha 410000, China; zjhseu@csust.edu.cn (J.Z.); lijue1207@stu.csust.edu.cn (J.L.); tqtimtdh@stu.csust.edu.cn (F.L.); pjh@stu.csust.edu.cn (J.P.)

**Keywords:** subgrade, C&D materials, permanent deformation, gray correlation, prediction model

## Abstract

Construction and demolition (C&D) materials obtained from the demolition of buildings are proven to be qualified and sustainable subgrade fillers. The permanent deformation response of subgrade C&D materials under different moisture contents, degrees of compaction, deviator stresses, and confining pressures was revealed by carrying out dynamic triaxial texts. Then, using a four-factor and three-level orthogonal test and by calculating the Gray correlation degree of each factor, the influence degree of each factor on the permanent deformation was determined. The results indicated that two different response types of the permanent deformation of subgrade C&D materials, plastic shakedown and plastic creep, were identified as reason behind the increase in stress levels. Also, according to the Gray correlation analysis results, the permanent deformation of highway subgrade filled with C&D materials is influenced by the deviator stress most significantly, followed by moisture content, degree of compaction, and confining pressure. Finally, a permanent deformation prediction model about this kind of subgrade filler with a reasonable prediction accuracy was proposed.

## 1. Introduction

With the rapid advancement of urbanization, many countries have vast waste resources, including plastics, glass shards, metal shavings, and construction and demolition (C&D) materials [1]. Survey results in some countries showed a large proportion of C&D materials in total waste [2]. The amount of C&D materials in the world is still growing, especially in China, where about 3.5 billion tons of C&D materials are produced every year, but the utilization rate is less than 5%. Compared to developed countries, e.g., 98% in Netherlands and 94% in Denmark [3], the recovery efficiency for C&D materials is extremely low in China. Most C&D materials are dumped in landfills or rural areas, bringing heavy burden to the environment and threatening human health [4]. With the increasing demands on the environment, excavation and quarrying are forbidden, resulting in the quality of natural road materials gradually decreasing [5]. Therefore, researchers carried out some tentative studies on the application of C&D materials in road engineering [1,6] as a kind of alternative materials for surface layers [7,8] and bases [9]. However, due to the lower strength of C&D materials, they can only partially replace the base or subbase aggregates; thus, there is still a large number of C&D materials can’t be effectively utilized in China. At the same time, since the average height of highway subgrade in China is about 3.4 m, generally higher subgrade makes the subgrade construction need more materials than surface layers and bases. By using C&D materials as subgrade fillers, the environmental pollution can be solved. Moreover, the shortage of road materials can be alleviated, and higher economic benefits can be obtained [10].

Excessive permanent deformation of highway subgrade is likely to lead to distresses such as rutting and cracking in the asphalt pavement [11,12]. Therefore, the permanent deformation characteristics of subgrade should be further studied. Great achievements in the permanent deformation of subgrade were achieved in the past decades. For example, Werkmeister et al. [13] and Chen et al. [14] revealed the plastic mechanical behavior of red clay, crushed stone, and peaty organic soil. Also, some scholars analyzed the influencing factors of permanent deformation of subgrade. By performing repeated load triaxial (RLT) tests on the coarse sand and silt under unconsolidated undrained conditions, Puppala et al. [15] found that permanent deformation is sensitive to the moisture content, degree of compaction, and deviator stress. Lin et al. [16] studied whether the permanent deformation of subgrade was related to the stress states by measuring the three-direction dynamic stresses of subgrade soils using an orthogonal earth pressure transducer. The monitoring data demonstrated that subgrade permanent deformation at a certain depth could be influenced by both confining pressure and deviator stress significantly. According to Gabon et al. [17], permanent deformation of three materials can be influenced by deviator stress, dry density, and moisture content significantly. Moreover, results of the RLT test by Azam et al. [18] on unbound materials showed that the moisture content and degree of compaction have an impact on permanent deformation. In general, four factors, confining pressure, deviator stress, degree of compaction, and moisture content, are discussed most frequently, whereas the study of the permanent deformation of subgrade fillers under different working conditions requires numerous specimens [19], which is time-consuming and costly. Wang et al. [20] and Fan [21] pointed out that influencing factors in practical engineering can be effectively determined by combining the orthogonal test and Gray correlation analysis. Moreover, the inherent properties of the system can be determined by the least test data.

After analyzing the influencing factors of permanent deformation, many researchers built various models to predict the permanent deformation and provide conveniences for engineering application. Initially, researchers proposed models of permanent deformation concerning load cycles. Barksdale found that the permanent deformation of silt and crushed stone showed a logarithmic growth trend with the increase of load cycles. He proposed the following half-logarithm model [22]:

(1) $$\varepsilon_{p}=\alpha_{1}+\alpha_{2}LnN$$

where *α*_1_ and *α*_2_ are the regression coefficients, and *N* is the number of load cycles.

Subsequently, based on the RLT test results of the silt, Monisimith et al. [23] proposed an exponential-type prediction model of permanent deformation with load cycles.

(2) $$\varepsilon_{p}=\alpha_{1}N^{\alpha_{2}}$$

where all parameters have the same meaning as Equation (1).

Then, some classical prediction models were proposed based on Equation (2). The model is only of mathematical meaning and does not roundly reflect the stress and physical state of subgrade fillers. A rational prediction model should embody these factors synchronously. Therefore, Puppala et al. introduced the bulk stress and the octahedral shear stress into Equation (2) successively in 1999 [24] and 2009 [25] to comprehensively considered the influence of stress state on permanent deformation.

(3) $$\varepsilon_{p}=\alpha_{1}N^{\alpha_{2}}{\left(\frac{\sigma_{oct}}{\sigma_{atm}}\right)}^{\alpha_{3}}$$

(4) $$\varepsilon_{p}=\alpha_{1}N^{\alpha_{2}}{\left(\frac{\sigma_{oct}}{\sigma_{atm}}\right)}^{\alpha_{3}}{\left(\frac{\tau_{oct}}{\sigma_{atm}}\right)}^{\alpha_{4}}$$

where *σ_atm_* is the reference stress (atmospheric intensity of pressure), *σ_atm_*= 100 kPa, *τ_oct_* is the octahedral shear stress, *σ_oct_* is the bulk stress, and *α*_1_ to *α*_4_ are the regression coefficients.

The two prediction models established by Puppala considered the influence of stress state but ignored the influence of physical factors. Later, Zhang [26] found that permanent deformation can be affected by the moisture state. Thus, the moisture conditions were taken into account in the prediction model to improve the Equation (4), and the model as shown in Equation (5) was established.

(5) $$\varepsilon_{p}=\alpha_{1}N^{\alpha_{2}}{\left(\frac{\sigma_{oct}}{\sigma_{atm}}\right)}^{\alpha_{3}}{\left(\frac{\tau_{oct}}{\sigma_{atm}}\right)}^{\alpha_{4}}{\left(\frac{w}{w_{omc}}\right)}^{\alpha_{5}}$$

where *w* is the actual moisture content of subgrade, *w_omc_* is the optimal moisture content of subgrade, and *α*_1_ to *α*_5_ are the regression coefficients.

Chow et al. [27] carried out RLT tests on 16 types of subgrade fillers to study the effects of shear stress and shear strength on permanent deformation. Finally, a model as shown in Equation (6) was proposed.

(6) $$\varepsilon_{p}=aN^{b}\sigma_{d}{}^{c}{\left(\frac{\tau_{f}}{\tau_{\max}}\right)}^{d}$$

where $\sigma_{d}$ is the deviator stress, $\tau_{f}$ is the shear stress, $\tau_{\max}$ is the shear strength, and *a*, *b*, *c*, and *d* are regression coefficients.

In previous studies, the elastic behavior of subgrade fillers under load was also considered in the prediction model of permanent deformation. For example, the model includes the resilient modulus established by Rasul et al. [28], which can fully reflect the complete mechanical behavior.

(7) $$\varepsilon_{p}=I\left(\frac{\sigma_{d}}{M_{r}}\right)N^{J}$$

where *σ_d_* is the deviator stress, *M_r_* is the resilient modulus, and *I* and *J* are the regression coefficients.

As mentioned above, the main factors affecting the permanent deformation of subgrades are the load cycles, the stress state, and the physical state. However, previous studies on comprehensively analyzing these factors together and comparing their levels of influence are less common and insufficient. At the same time, in the prediction models shown in Equations (1)–(7), the effect of the degree of compaction on permanent deformation was not visibly reflected. Therefore, it is a challenge and meaningful to comprehensively analyze the impact of various factors and propose a straightforward and applicable model for predicting the permanent deformation of C&D materials.

As a potential subgrade filler, the permanent deformation is an important evaluation index for C&D materials. At present, few pieces of literature report the permanent deformation of the subgrade filled with C&D materials. This study aims to reveal the permanent deformation response of C&D materials under repeated loads, investigate the influence of various factors on their permanent deformation, and finally propose a prediction method for permanent deformation of the subgrade built by C&D materials. The structure of this paper is as follows: the preparation process and the basic properties of the C&D materials are introduced in the forthcoming section. After that, an orthogonal test was designed to comprehensively investigate various factors and levels, and dynamic triaxial tests were carried out. Afterward, the Gray correlation degree of each factor was calculated to determine different influence degrees on the permanent deformation. Then, the applicability of some existing models to predict the permanent deformation of C&D materials was confirmed by data from the orthogonal test in this study. Next, based on the results of Gray correlation analysis, the existing model with good applicability was improved, and the new model for predicting the permanent deformation of C&D materials was verified for accuracy. The main findings are summarized in the last section.

## 2. Test Program

### 2.1. Material Preparation and Basic Properties Tests

**Figure 1:**
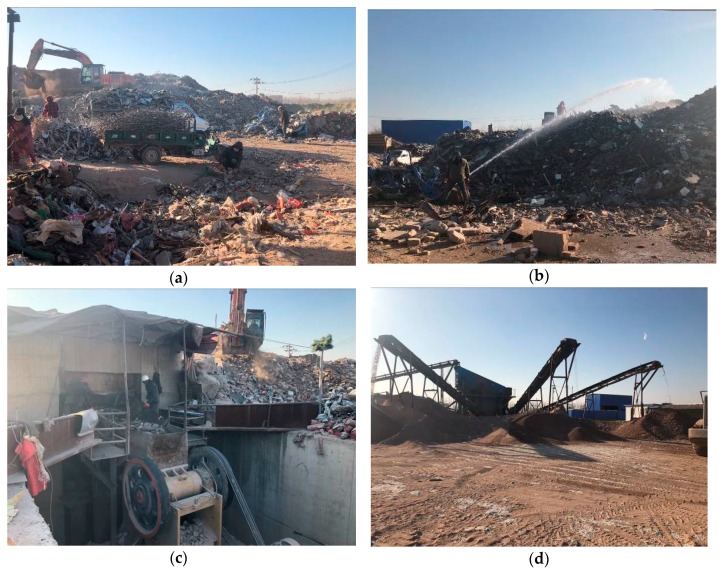
Subgrade filler preparation process: (**a**) eliminating clutter; (**b**) sprinkling; (**c**) crushing; (**d**) screening.

**Figure 2:**
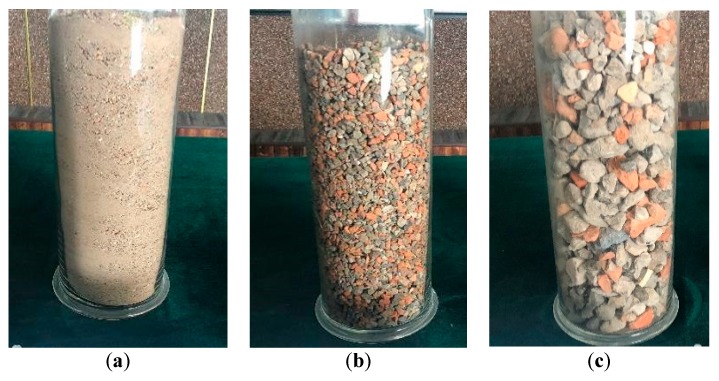
Construction waste of different particle size ranges after sieving: (**a**) 0–4.75 mm; (**b**) 4.75–9.5 mm; (**c**) 9.5–31.5 mm.

The C&D materials were taken from the Tongzhou–Daxing section of the capital area highway loop project located in Beijing, China. The preparation process of this subgrade fillers was as follows (Figure 1): firstly, plastic, steel rebar, wastepaper, and household garbage were removed from raw materials. Then, raw materials were sprinkled to prevent excessive dust from being generated in the crushing process and polluting the environment. After sprinkling, the jaw crusher was used for crushing raw materials immediately. Finally, the crushed construction waste was screened into materials with various particle sizes (Figure 2).

**Figure 3:**
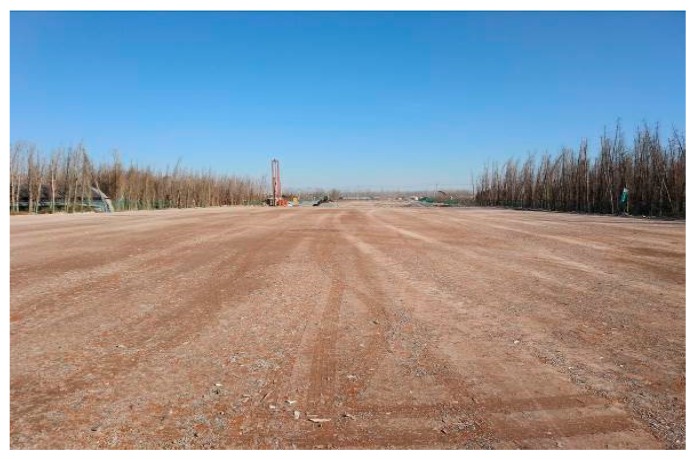
Highway subgrade filled with construction and demolition (C&D) materials.

According to field test results based on different mixing proportions, three batches of materials with particle sizes ranging from 0 to 4.75 mm, 4.75 to 9.5 mm, and 9.5 to 31.5 mm were mixed to form subgrade fillers in a mass ratio of 25%:25%:50%, so that the particles of different batches could be better interlocked to ensure higher strength of subgrade. Figure 3 shows the highway subgrade filled with these kinds of materials.

**Figure 4:**
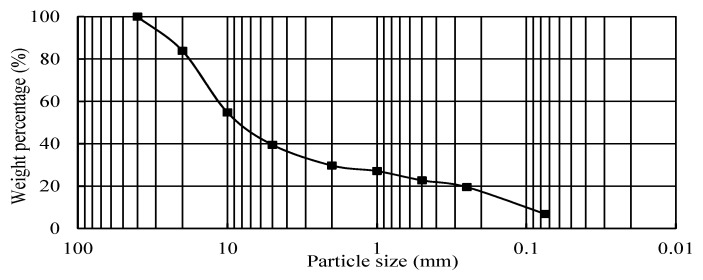
Gradation curve of the used C&D materials.

The basic properties were tested by selecting some representative C&D materials at the construction site. The plastic limit was 22.5%, the liquid limit was 28.1%, and the natural moisture content was 12.7%. The compaction test results indicated that the maximum dry density was 1.843 g/cm^3^, and the optimal moisture content (OMC) was 14.8%. The contents of organic and soluble salt were 1.90% and 0.376%, respectively. The values of CBR corresponding to 30, 50, and 98 times of compaction for each layer were 10.9%, 47.7%, and 98.6%, respectively. The particle size analysis for these materials is presented in Figure 4.

**Figure 5:**
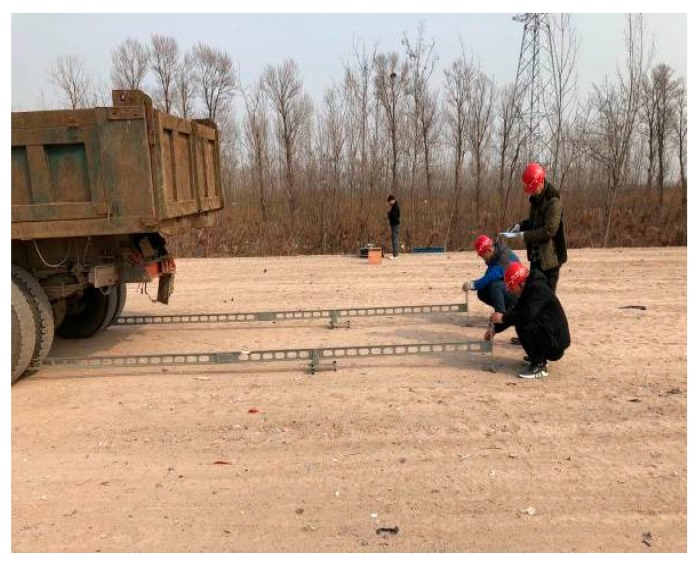
Deflection value field test.

In China, the holistic bearing capacity of highway subgrade is evaluated according to the deflection value. In this study, the six continuous sections in the experiment section (K12 + 960–K13 + 060) were selected to measure deflections of the completed subgrade top by Beckman beam deflection tests (JTG E60-2008). The truck used in the test had a load of 100 kN and a tire pressure of 0.7 MPa, and test points were located at the centerline of subgrade. Figure 5 and Table 1 respectively show the test process and results.

Table 1 shows that the deflection values of these sections were 157–205 (0.01 mm), which are smaller than the design deflection value of 232 (0.01 mm). Thus, the highway subgrade filled with C&D materials meets the requirements of the standard in China. Even so, the permanent deformation characteristics of subgrade filled with C&D materials also needed to be further studied since a larger permanent deformation would decrease the road life.

### 2.2. Test Plan of the Permanent Deformation

The permanent deformation characteristics of C&D materials were studied by dynamic triaxial tests. As mentioned above, four influencing factors were set as factor A (moisture content), factor B (degree of compaction), factor C (confining pressure), and factor D (deviator stress). Usually, the moisture content in subgrades will gradually increase and become stable finally [29]. Elliott [30] conducted field tests on the moisture content of subgrade, and the results showed that the actual moisture content of subgrade after completing two years was about 1.2 times that of the OMC. Zhang et al. [31] investigated subgrade of some expressways in China and found that the actual moisture content of subgrade was 10% to 20% higher than the OMC after about 20 years of operation. On this basis, combined with the climate characteristics of less rain in northern China, three moisture contents, i.e., 0.9 OMC, 1.0 OMC, and 1.1 OMC, were selected to cover the possible moisture contents of subgrade. In order to ensure that degrees of compaction set in this study fully covered the range required by the subgrade design standards (JTG D30-2015), three degrees of compaction were set as 96%, 93%, and 90%. Uzan [32] calculated the compressive stress at the top of subgrade with a pavement structure thickness of 50–80 cm (including the commonly used thicker or thinner pavement types) in Israel and found that the values were distributed in the range of 37–54 kPa or 12–21 kPa depending on the pavement structure. Cong et al. [33] calculated the asphalt pavement structure commonly used in China and pointed out that subgrade surface compressive stress was around 50 kPa and the lateral stress of fillers was approximately 28 kPa. Then, 28 kPa was taken as the constant confining pressure, and 28 kPa, 48 kPa, and 69 kPa were determined as three deviator stress levels in his study. Zhao et al. [34] tested the permanent deformation of subgrade soil samples with different confining pressures (0 kPa, 21 kPa, and 42 kPa), and found that confining pressure was not the key factor affecting the permanent deformation; he proposed that 21 kPa could represent the confining pressure of most subgrade soil. Based on these findings, combined with the investigation results obtained by our research group, the deviator stress levels were set at 28 kPa, 48 kPa, and 69 kPa, and the confining pressure levels were set at 12 kPa, 28 kPa, and 42 kPa to simulate the stress state of Chinese subgrade as accurately as possible. Different factors at different levels were combined to simulate different working conditions, and the specific combination mode is listed in Table 2. Such a multi-factor and multi-level arrangement is called an orthogonal test design, which has the characteristics of uniform dispersion and good comparability [20]. Furthermore, the orthogonal test can shorten the test period by reducing test times [35]. Under such advantages, the orthogonal test is applied to various fields [36]. 

### 2.3. Test Process of the Permanent Deformation

**Figure 6:**
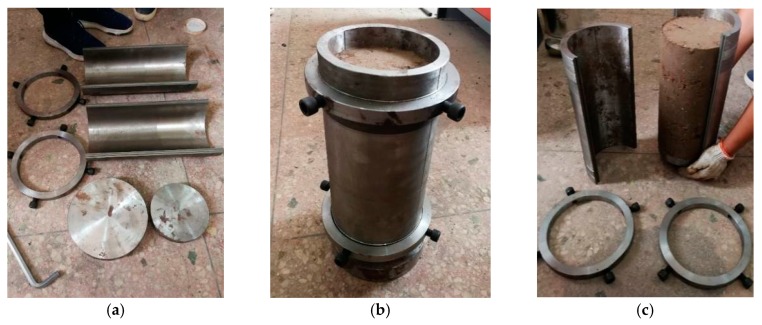
Forming mold: (**a**) components; (**b**) molding; (**c**) demolding.

**Figure 7:**
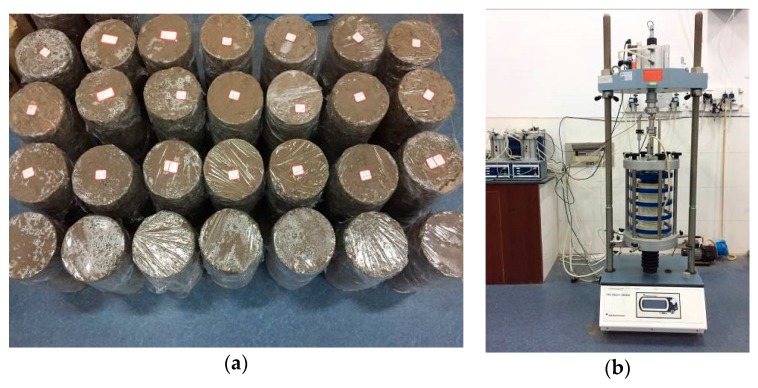
Completed specimens and dynamic triaxial apparatus: (**a**) specimens; (**b**) apparatus.

According to requirements for the specimen size in the dynamic triaxial test and the range of particle size of C&D materials, cylindrical specimens with a height of 300 mm and a diameter of 150 mm were prepared. Different moisture contents and degrees of compaction of specimens were set according to Table 2. The self-developed forming mold was used (Figure 6) to avoid the specimen being affected by non-negligible disturbances. The five layers of the specimens were filled and compacted by a metal bar. Afterward, we compacted the specimen to the designed height, which corresponded to the degree of compaction designed. After the specimen was finished, it was sealed with a freshness-keeping film to prevent water evaporation. The shaped specimens are shown in Figure 7a.

Dynamic triaxial tests were carried out after specimens were completed. The selected apparatus was the Dynatriax 100/14 static–dynamic triaxial test system made by the Italian Controls company (Milan, Italy) (Figure 7b). The cyclic load with a half-sine load pulse, which had the rest period of 0.8 s and the duration of 0.2 s, was used following NCHRP1-28A. Before formal load, the initial plastic deformation was eliminated by applying 2000 cycles of pre-loads to specimens, and the stress level selected for pre-loads was same as that for the formal load. Then, the data for the 10,000th cycle were taken as the final permanent deformation value of the specimens [37]. Replicable tests were carried out for every test number included in Table 2. If the error for each replicable test result did not exceed ±5%, the test results were adopted since they were deemed reliable.

## 3. Results and Discussion

### 3.1. Permanent Deformation Response

The evolution of permanent deformation of subgrade fillers with load cycles is complex, and the response of the material under cyclic loads can be classified into four different categories according to Werkmeister et al. [38]:

(1) Elastic shakedown, where the applied cyclic stress is sufficiently small compared to the strength of the material, and the material response is purely elastic.
(2) Plastic shakedown, where the rate of cumulative permanent deformations decreases gradually until it reaches a stabilized state, and the material gradually achieves a long-term steady-state response.
(3) Plastic creep, where the permanent deformation continues to accumulate, and the material response to cyclic loads shows an unstable state; however, the material plastic response neither terminates nor leads to a sudden collapse.
(4) Incremental collapse, where the applied cyclic stress is relatively large to cause the material to reach and exceed the yield stress, and the accumulated plastic deformations then increase rapidly with failure occurring in a small number of load cycles.

**Figure 8:**
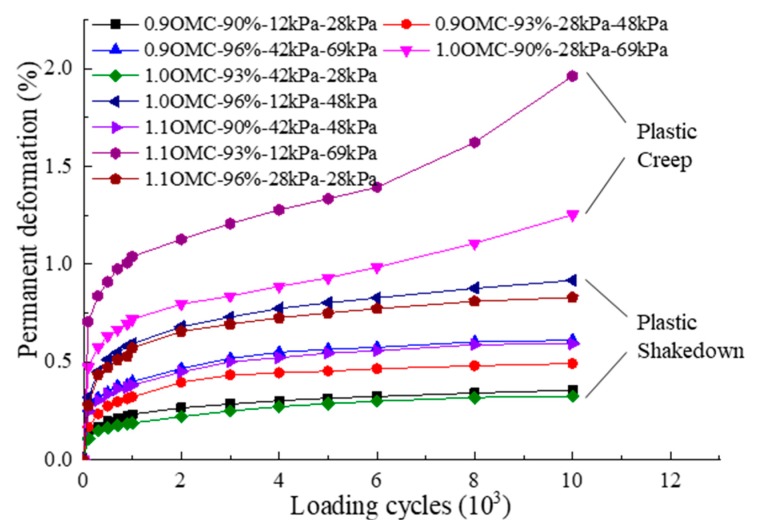
Permanent deformation of C&D material specimens at different working conditions.

The permanent deformation under different conditions in Table 2 is shown in Figure 8. The graphical interpretations in the figure are arranged in the order of moisture content–degree of compaction–confining pressure–deviator stress. Based on the above-stated definitions of different deformation stages, two states of plastic shakedown and plastic creep can be observed. Moreover, all specimens showed the permanent deformation at different degrees; thus, none of results showed behavior in the elastic shakedown range. As an example, at a relatively safe stress level (1.0 OMC, 93% degree of compaction, 42 kPa confining pressure, 28 kPa deviator stress), after a finite number of load cycles, the specimen exhibited a long-term plastic shakedown state with no more accumulation of the permanent deformation. This was because, at the initial stage of loads, the air and part of the water in the pores of the specimen were squeezed, improving the bonding of C&D materials particles and the ability of the specimen to resist lower cyclic loads under higher confining pressure. Similarly, another relatively dangerous condition (1.1 OMC, 93% degree of compaction, 12 kPa confining pressure, 69 kPa deviator stress) was analyzed. Upon increasing deviator stress and decreasing confining pressure, the type of permanent deformation response to cyclic loads changed from a stable state to an unstable state. That is, at a higher stress level, the specimen showed a plastic creep state. This phenomenon was most likely due to the fact that, after the compression deformation of the specimen tended to be stable, the higher deviator stress caused some partial shear deformations to occur in some areas inside the specimen and they developed gradually, resulting in continuous accumulation of the permanent deformation.

In conclusion, the permanent deformation response of C&D materials exhibited a stress-dependent characteristic. It is worth mentioning that no obvious incremental collapse state was observed in the permanent deformation response of C&D materials; this infers that subgrade filled with C&D materials should have better stability and durability in the service period.

### 3.2. Gray Correlation Analysis of Influencing Factors

Table 3 lists the final permanent deformation amounts of all specimens in the design of the orthogonal test. According to the test results, the permanent deformation amount varied with different combinations of factors and levels. As shown in Table 3, the maximum permanent deformation amount was 1.912% (moisture content 1.1 OMC, deviator stress 69 kPa, degree of compaction 93%, and confining pressure 12 kPa), and the minimum permanent deformation amount was close to 0.33% (moisture content OMC, deviator stress 28 kPa, degree of compaction 93%, and confining pressure 42 kPa). However, the extreme value of permanent deformation did not occur under the maximum or minimum levels of each factor set as Table 2. The permanent deformation of C&D materials was influenced by all factors to different degrees. For instance, when deviator stress was 28 kPa, there was a smaller permanent deformation amount; when deviator stress increased to 48 kPa or 69 kPa, the permanent deformation amount became larger generally. The minimum and maximum permanent deformation were 0.329% (deviator stress = 28 kPa) and 1.912% (deviator stress = 69 kPa), respectively. In the same way, moisture content and degree of compaction could influence the permanent deformation of C&D materials significantly. Therefore, it was necessary to determine the factor with the most significant influence.

Gray correlation analysis was used to determine the different influence degrees of various factors on dependent variables in complex systems. The geometric proximity between discrete sequences can be evaluated by the Gray correlation model [20]. In the present study, the key influencing factors of the permanent deformation characteristics of C&D materials were determined according to the geometric proximity. The Gray relational order and the Gray relational degree were determined by the following steps:

(1) The data series $X_{i}=\left\{x_{i}\left(k\right),k=1,2,\ldots n\right\}$ was taken as the comparison sequence, and $X_{0}=\left\{x_{0}\left(k\right),k=1,2,\ldots n\right\}$ was taken as the reference sequence.

(2) A discrete function of the relational degree coefficient (the Gray relational coefficient) was obtained by the following equation so that the relational degree between the comparison and reference sequences could be determined:

(8) $$\xi_{i}\left(k\right)=\frac{\left[\min_{i}\min_{k}\left|X_{i}\left(k\right)-X_{0}\left(k\right)\right|+\rho \max_{i}\max_{k}\left|X_{i}\left(k\right)-X_{0}\left(k\right)\right|\right]}{\left[\left|X_{i}\left(k\right)-X_{0}\left(k\right)\right|+\rho \max_{i}\max_{k}\left|X_{i}\left(k\right)-X_{0}\left(k\right)\right|\right]}$$

where

(9) $$\Delta_{\max}=\underset{i}{\max}\underset{k}{\max}\left\{\Delta_{0i}\left(k\right)\right\}\left(i=1,2,\ldots ,m;k=1,2,\ldots ,n\right)$$

(10) $$\Delta_{\min}=\underset{i}{\min}\underset{k}{\min}\left\{\Delta_{0i}\left(k\right)\right\}\left(i=1,2,\ldots ,m;k=1,2,\ldots ,n\right)$$

are the maximum and minimum proximities, and *ρ* is the coefficient distinguishing the proximity degree of *X*_0_ and *X_i_*.

(3) The Gray relational degree of the comparison sequence *X_i_* to the reference sequence *X*_0_ was obtained by

(11) $$\gamma_{i}=\frac{1}{n}\sum_{i=1}^{n}\xi_{i}\left(k\right)$$

Table 3 lists influence factors (moisture content, degree of compaction, confining pressure, deviator stress) and final permanent deformation amounts, which were taken as the comparison sequence and the reference sequence, respectively. The equivalence of all factors was ensured by processing the primary data. In Table 3, a new data sequence (Table 4) was generated by dividing the initial values of rows 2–9 by the initial values of the first row, as shown in Equation (12).

(12) $$X_{i}\left(k\right)=\frac{x_{i}\left(k\right)}{x_{i}\left(1\right)}$$

After initiating treatment, the results are listed in Table 4.

The absolute difference between the two sequences was acquired by

(13) $$\Delta_{i}\left(k\right)=\left|X_{i}\left(k\right)-X_{0}\left(k\right)\right|$$

Table 5 shows the approach degree results.

The Gray correlation coefficient was given by

(14) $$\xi_{i}\left(k\right)=\frac{\Delta_{\min}+\rho \Delta_{\max}}{\Delta_{i}\left(k\right)+\rho \Delta_{\max}}$$

where *ρ* = 0.5 [20]. From Table 5, *∆*_max_ = 4.3557 and *∆*_min_ = 0.

Table 6 shows the correlation coefficient results.

Finally, Equation (11) was used to calculate the Gray correlation degree of the reference sequence *X*_0_ and the comparison sequence *X*_i_. The calculation results were as follows:

*γ*_1_ = 0.7151, *γ*_2_ = 0.7081, *γ*_3_ = 0.6443, *γ*_4_ = 0.7770.

The order of the Gray correlation was Factor D > Factor A > Factor B > Factor C (deviator stress > moisture content > degree of compaction > confining pressure). Therefore, the influence of deviator stress on the permanent deformation of C&D materials was the most significant. Secondly, the effects of the moisture content and degree of compaction were similar because the Gray correlation degrees were approximate. It also could be found that the Gray correlation of confining pressure was slightly smaller than the other three factors; thus, the confining pressure had the least influence on the permanent deformation of C&D materials in this study.

### 3.3. Prediction Model of Permanent Deformation

Some models were selected for reference and comparison to establish a model for predicting the permanent deformation of C&D materials. By analyzing the prediction models mentioned above (Equations (1)–(7)), it can be seen that the first five models made it easy to obtain model parameters and the model form was simple and easy to be understood. Therefore, Equations (1)–(5) were selected in this study to determine the applicability of predicting the permanent deformation of C&D materials under different working conditions. Table 7 shows the obtained regression coefficients and evaluation indexes of Equations (1)–(5) by permanent deformation values of specimens under the nine different working conditions shown in Table 2, which were subjected to 1, 100, 300, 500, 700, 900, 1000, 2000, 3000, 4000, 5000, 6000, 8000, and 10,000 load cycles. According to the value of the determination coefficient (*R*^2^), Witczak et al. [39] put forward the subjective criteria for prediction. A fair fit was defined as 0.4 ≤ *R*^2^ ≤ 0.69, a good fit is *R*^2^ = 0.7–0.89, and an excellent fit is *R*^2^ ≥ 0.9. Zhang et al. [40] also used this standard to evaluate the fitting effect of the prediction model for the dynamic resilient modulus of subgrade soil.

Table 7 demonstrates that the values of adjusted *R*^2^ for the first three equations were all lower than 0.4, which shows that these models were not applicable to describe the permanent deformation of C&D materials. The adjusted *R*^2^ values of Equations (4) and (5) were between 0.7 and 0.89 and root-mean-square errors were also significantly reduced. Although the prediction accuracy was increased encouragingly, this study still expects a better improvement.

Based on the Gray correlation analysis, it was found that the degree of compaction had a non-negligible influence on the permanent deformation. However, Equations (4) and (5) did not consider the contribution of the degree of compaction to permanent deformation. Therefore, the form of Equation (5) was revised, and the degree of compaction reflecting the physical state of the material was taken into account as a new variable, and the new model was established as shown in Equation (15).

(15) $$\varepsilon_{p}=\alpha_{1}N^{\alpha_{2}}{\left(\frac{\sigma_{oct}}{\sigma_{atm}}\right)}^{\alpha_{3}}{\left(\frac{\tau_{oct}}{\sigma_{atm}}\right)}^{\alpha_{4}}{\left(\frac{w}{w_{omc}}\right)}^{\alpha_{5}}K^{\alpha_{6}}$$

where *K* represents the degrees of compaction of subgrade, and *α*_6_ is the regression coefficient.

The major influence factors, including the physical state, the constraint effect, and shear effect, and the load cycles were comprehensively reflected in the model. Therefore, the prediction model could be better understood. Moreover, the universal triaxial testing system and basic performance test were enough to get its parameters. The parameters and goodness of this model are shown in Table 8.

Table 8 shows that the new model had an adjusted *R*^2^ value greater than 0.9 and the root-mean-square error remained at a low level. The new model provided an excellent fitting and was more suitable than Equations (4) and (5) for predicting the permanent deformation of C&D materials. In order to ensure the robustness of the new model, verifications were implemented via the permanent deformation data of C&D materials under different numbers of loads with the other nine different conditions, as shown in Table 9 and a research report by Azam et al. [18]. These working conditions comprehensively covered a range of research levels of various influencing factors and could accurately estimate the precision of the new prediction model.

**Figure 9:**
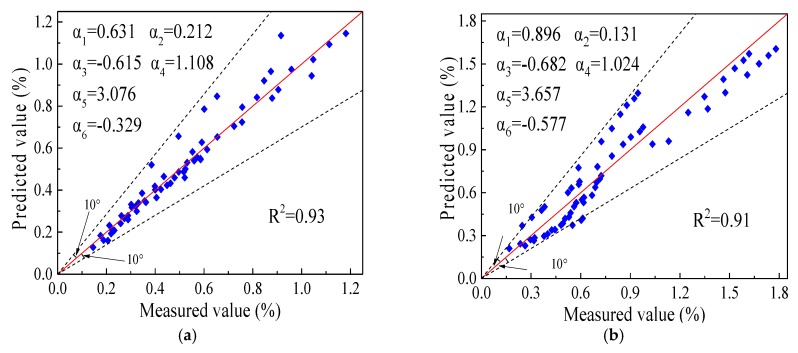
Predicted versus measured permanent deformation values for verifying the new model: (**a**) this study (Table 9); (**b**) test data of Azam et al.

Figure 9 shows the measured values and predicted values used to verify the new model, which were all close. Thus, the permanent deformation of subgrade filled with C&D materials could be adequately predicted by the new model.

## 4. Conclusions

In this study, material basic properties tests and dynamic triaxial tests were carried out for C&D materials from northern China. The permanent deformation response of these materials under repeated loads was analyzed. Then, the different influence degrees of deviator stress, confining pressure, degree of compaction, and moisture content on the permanent deformation were determined according to the Gray correlation analysis. Finally, a new permanent deformation prediction model that comprehensively considers the stress state and physical state of the subgrade filler was established and verified. The following conclusions were drawn:

(1) The permanent deformation response of C&D materials exhibits a stress-dependent characteristic. Upon increasing in stress levels, the permanent deformation response of C&D materials changed from plastic shakedown to plastic creep in this study.

(2) The results of the Gray correlation analysis indicated that the deviator stress influences the permanent deformation of highway subgrade filled with C&D materials most significantly, followed by moisture content, degree of compaction, and confining pressure. However, the Gray correlation degrees of these factors are relatively close. Therefore, when analyzing the permanent deformation problem, the influence of these factors should be considered comprehensively.

(3) According to the Gray correlation analysis, the permanent deformation of C&D materials is significantly influenced by the degree of compaction. Therefore, a new exponential model including load cycles, moisture content, degree of compaction, volume stress, and shear stress was established. The results showed that the proposed model, which has a high fitting degree, can provide reference for the prediction of the permanent deformation of highway subgrade filled with C&D materials.

## Figures and Tables

**Table 1:** Test results of deflection value.

Section	K12 + 980	K12 + 990	K13 + 000	K13 + 010	K13 + 020	K13 + 030
Deflection value (0.01 mm)	177	183	205	185	189	157

**Table 2:** Dynamic triaxial test plan. OMC—optimal moisture content.

Test Number	Factor A, Ratio of Moisture Content to OMC	Factor B, Degree of Compaction (%)	Factor C, Confining Pressure (kPa)	Factor D, Deviator Stress (kPa)
1	0.9	90	12	28
2	0.9	93	28	48
3	0.9	96	42	69
4	1.0	90	28	69
5	1.0	93	42	28
6	1.0	96	12	48
7	1.1	90	42	48
8	1.1	93	12	69
9	1.1	96	28	28

**Table 3:** Dynamic triaxial test results of construction and demolition (C&D) materials.

No.	Test	Final Permanent Deformation (%)
1	MC 0.9 OMC, C 90%, CP 12 kPa, DS 28 kPa	0.357
2	MC 0.9 OMC, C 93%, CP 28 kPa, DS 48 kPa	0.492
3	MC 0.9 OMC, C 96%, CP 42 kPa, DS 69 kPa	0.612
4	MC 1.0 OMC, C 90%, CP 28 kPa, DS 69 kPa	1.253
5	MC 1.0 OMC, C 93%, CP 42 kPa, DS 28 kPa	0.329
6	MC 1.0 OMC, C 96%, CP 12 kPa, DS 48 kPa	0.914
7	MC 1.1 OMC, C 90%, CP 42 kPa, DS 48 kPa	0.593
8	MC 1.1 OMC, C 93%, CP 12 kPa, DS 69 kPa	1.912
9	MC 1.1 OMC, C 96%, CP 28 kPa, DS 28 kPa	0.842

MC—moisture content, C—degree of compaction, CP—confining pressure, DS—deviator stress.

**Table 4:** Data after initiating treatment.

*X*_1_	*X*_2_	*X*_3_	*X*_4_	*X*_0_
1.0000	1.0000	1.0000	1.0000	1.0000
1.0000	1.0333	2.3333	1.7143	1.3782
1.0000	1.0667	3.5000	2.4643	1.7143
1.1111	1.0000	2.3333	2.4643	3.5098
1.1111	1.0333	3.5000	1.0000	0.9216
1.1111	1.0667	1.0000	1.7143	2.5602
1.2222	1.0000	3.5000	1.7143	1.6611
1.2222	1.0333	1.0000	2.4643	5.3557
1.2222	1.0667	2.3333	1.0000	2.3585

**Table 5:** Approach degree calculation results.

*∆*_1_	*∆*_2_	*∆*_3_	*∆*_4_
0.0000	0.0000	0.0000	0.0000
0.3782	0.3448	0.9552	0.3361
0.7143	0.6476	1.7857	0.7500
2.3987	2.5098	1.1765	1.0455
0.1895	0.1118	2.5784	0.0784
1.4491	1.4936	1.5602	0.8459
0.4388	0.6611	1.8389	0.0532
4.1335	4.3224	4.3557	2.8915
1.1363	1.2919	0.0252	1.3585

**Table 6:** Correlation degree calculation results.

*ξ*_1_	*ξ*_2_	*ξ*_3_	*ξ*_4_
1	1	1	1
0.8710	0.8811	0.7278	0.8837
0.7815	0.7977	0.5886	0.7730
0.5157	0.5044	0.6847	0.7096
0.9309	0.9581	0.4977	0.9702
0.6380	0.6310	0.6208	0.7512
0.8534	0.7944	0.5814	0.9796
0.3819	0.3714	0.3697	0.4690
0.6921	0.6641	0.9902	0.6528

**Table 7:** Regression coefficients and model goodness.

Prediction Model	*α*_1_	*α*_2_	*α*_3_	*α*_4_	*α*_5_	*R* ^2^	adj-*R*^2^	RMSE	Correlation
$\varepsilon_{p}=\alpha_{1}+\alpha_{2}LnN$	−0.221	0.106	—	—	—	0.24	0.23	0.31	Inferior
$\varepsilon_{p}=\alpha_{1}N^{\alpha_{2}}$	0.115	0.208	—	—	—	0.24	0.23	0.29	Inferior
$\varepsilon_{p}=\alpha_{1}N^{\alpha_{2}}{\left(\frac{\sigma_{oct}}{\sigma_{atm}}\right)}^{\alpha_{3}}$	0.106	0.209	−0.089	—	—	0.25	0.24	0.30	Inferior
$\varepsilon_{p}=\alpha_{1}N^{\alpha_{2}}{\left(\frac{\sigma_{oct}}{\sigma_{atm}}\right)}^{\alpha_{3}}{\left(\frac{\tau_{oct}}{\sigma_{atm}}\right)}^{\alpha_{4}}$	0.514	0.212	−0.908	1.612	—	0.75	0.74	0.17	Good
$\varepsilon_{p}=\alpha_{1}N^{\alpha_{2}}{\left(\frac{\sigma_{oct}}{\sigma_{atm}}\right)}^{\alpha_{3}}{\left(\frac{\tau_{oct}}{\sigma_{atm}}\right)}^{\alpha_{4}}{\left(\frac{w}{w_{omc}}\right)}^{\alpha_{5}}$	0.331	0.212	−0.603	1.106	3.101	0.89	0.88	0.11	Good

Notes: adj-*R*^2^ is the adjusted *R*^2^; RMSE is the root-mean-square error.

**Table 8:** Regression coefficients and goodness of the new model.

*α*_1_	*α*_2_	*α*_3_	*α*_4_	*α*_5_	*α*_6_	*R* ^2^	adj-*R*^2^	RMSE	Correlation
0.631	0.212	−0.615	1.108	3.076	−0.329	0.92	0.91	0.11	Excellent

**Table 9:** Test plan for verifying the new model.

Test Number	Ratio of Moisture Content to OMC	Degree of Compaction (%)	Confining Pressure (kPa)	Deviator Stress (kPa)
1	0.9	96	28	28
2	1.0	96	42	69
3	1.1	96	12	48
4	0.9	93	12	28
5	1.0	93	28	69
6	1.1	93	42	48
7	0.9	90	28	48
8	1.0	90	12	28
9	1.1	90	42	69
